# The role of transition dipole phase in atomic attosecond transient absorption from the multi-level model

**DOI:** 10.1063/1.5124441

**Published:** 2019-10-15

**Authors:** Guanglu Yuan, Shicheng Jiang, Ziwen Wang, Weijie Hua, Chao Yu, Cheng Jin, Ruifeng Lu

**Affiliations:** 1 Department of Applied Physics, Nanjing University of Science and Technology, Nanjing, Jiangsu 210094, People's Republic of China; 2 State Key Laboratory of Transient Optics and Photonics, Xi'an Institute of Optics and Precision Mechanics, Chinese Academy of Sciences, Xi'an, Shaanxi 710119, People's Republic of China; 3 State Key Laboratory of Molecular Reaction Dynamics, Dalian Institute of Chemical Physics, Chinese Academy of Sciences, Dalian, Liaoning 116023, People's Republic of China

## Abstract

Based on a multilevel model considering enough bound electronic states of atoms, we theoretically study the role of the transition dipole phase (TDP) in the attosecond transient absorption (ATA) spectrum of helium in intense laser fields. By solving the stationary Schrödinger equation with B-spline basis sets, we first calculate the transition dipole moments with well-defined phases between the bound states. Using the modified multilevel model, we reveal that the TDP plays an important role in determining the spectral structures if two or more paths populate the excited states from the ground state. Our multilevel model with the accurate TDP is convenient to address the origin of atomic ATA spectral structures by freely removing or adding specific electronic states and has been justified by comparing with the ATA spectra via directly solving the time-dependent Schrödinger equation. Hopefully, further incorporating macroscopic propagation into the model will provide indepth physical insights into experimental ATA spectra.

## I. INTRODUCTION

From the interaction of an intense focused laser with a gaseous medium, the generated high harmonics have been established as a tabletop light source to produce the isolated attosecond pulse or the attosecond pulse train in the extreme ultraviolet (XUV).^1–7^ Such an attosecond XUV field can be precisely synchronized with a moderate infrared (IR) laser to offer a scheme for the so-called attosecond transient absorption (ATA) spectroscopy in a time-resolved way to trace the rapid electronic dynamics at the sub-optical-cycle scale. The ATA spectrum is a fully optical method, using a femtosecond IR pulse to dress the system so that the spectrum of an attosecond XUV pulse transmitted through a sample and recorded as a function of time delay with respect to the IR pulse is modified, providing the information of absorption and emission of light. This technique has less disturbance to the target system than the detection of charged particles, and so the experimental signal-to-noise ratio is higher. It has been successfully applied to rare atoms and simple molecules, such as krypton, argon, and helium atoms^8–19^ and hydrogen, oxygen, and nitrogen molecules,^20–25^ and has also been employed in solid targets.^26–29^ For atomic systems, the ATA spectra have displayed several interesting features including the light-induced states (LISs),^30,31^ Autler-Townes splitting,^32,33^ hyperbolic sidebands associated with perturbed free-induction decay,^34^ and AC Stark shift of the absorption line,^35^ indicating various physical processes motivated by the electronic motion.

To address the experimentally measured ATA spectra, usually two parts are important in theoretical simulations. (i) The single-atom induced dipole by the combined XUV and IR pulses, which can be calculated by solving the time-dependent Schrödinger equation (TDSE), and (ii) the macroscopic response to the entire nonlinear medium, obtained by solving the three-dimensional Maxwell's wave equations for both the XUV and IR fields. In this work, we will restrict ourselves to the first part for introducing the theoretical method at the single-atom level, which considers the atomic system with one active electron and its excitation below the ionization threshold. The macroscopic propagation will be deferred to a subsequent work for direct comparison with experiments.

At present, the most accurate method to obtain a single-atom induced dipole is *ab initio* calculations by numerically solving the TDSE. However, this approach is very time-consuming and is not easy to decode the spectral information. It is necessary to develop other models to clearly identify and interpret desired characteristics in the ATA spectra. Meanwhile, it is worth speeding up the calculation of the single-atom response to feed into the propagation equations, in which typically hundreds of induced dipoles are needed. To date, a well-known three-level model has been widely used to reproduce some pronounced features in the ATA spectra reasonably well,^33,36–41^ and a few analytical theories have been proposed based on this model.^32,33,40^ In fact, the spectral width of the XUV pulse is wide enough to cover many excited bound states in the interaction process; thus, the three-level model should be extended to the multilevel one to include more electronic states involved in ATA. A few attempts have been carried out so far^11,38,40,42,43^ to explain the experimental absorption spectra or to compare with the spectra by the three-level model. The careful calibration of the multilevel model with the TDSE has not been completed. In the multilevel model, and in the three-level model as well, the key ingredients are the transition dipole moments (TDMs) between the bound states, which are usually obtained through the data of oscillation strength in the literature;^44,45^ nevertheless, the plus or minus sign of TDM cannot be determined. Is this sign (or phase) important for calculating the ATA spectra? And how could the phase of TDM be defined? Some of the recent works indeed show the significant role of the transition dipole phase (TDP) in solid high harmonic generation by solving the semiconductor Bloch equations.^46,47^ Motivated by these open questions and reported studies, we will explore the effect of the transition dipole phase on the atomic ATA processes.

In this work, we use an alternative way to obtain the TDMs by numerically solving the time-independent Schrödinger equation with the B-spline expansion technique, in which their phases can be well defined. Then, the TDMs are inserted into the multilevel model to precisely calculate the single-atom ATA spectra. Our main focus is to examine the role of the transition dipole phase (TDP) in ATA calculations. This paper is structured as follows. In Sec. II, we introduce our theoretical methods including the calculations of TDMs and the single-atom absorption spectrum with the multilevel model. In Sec. III, we take the helium atom as a benchmark atom system to discuss how the TDP plays a role in ATA. This paper will be concluded in Sec. IV.

## II. THEORETICAL METHODS

An intuitive and practical theoretical method for calculating the atomic ATA spectrum under a moderately strong IR laser field and a delayed attosecond XUV pulse is proposed in this section. This method is based on the single-active-electron (SAE) approximation and considers linearly polarized laser fields where the IR and XUV polarization vectors are parallel. The target atom is helium. Atomic units are used unless otherwise indicated.

### A. Transition dipole moment calculated with the B-spline basis set

The stationary Schrödinger equation for an outmost electron of atom can take the form

$$[-\frac{1}{2}\nabla^{2}+V(r)]\psi (\vec{r})=E\psi (\vec{r}),$$ (1)

where *V*(*r*) is the one-electron model potential. In order to solve Eq. (1), we use the B-spline function to form the basis set. For the order *k* and a knot sequence on the *r* axis $\left\{r_{1}\leq r_{2}\leq \cdots \leq r_{N}\leq \cdots \leq r_{N+k}\right\}$, the B-spline function is defined as

$$B_{i,1}(r)=\{\begin{array}{cc} 1, & r_{i}\leq r<r_{i+1},\\ 0, & \text{otherwise}, \end{array}$$ (2)

$$B_{i,k}(r)=\frac{r-r_{i}}{r_{i+k-1}-r_{i}}B_{i,k-1}(r)+\frac{r_{i+k}-r}{r_{i+k}-r_{i+1}}B_{i+1,k-1}(r).$$ (3)

Due to the central symmetry of the potential *V*(*r*), the eigenfunction of Hamiltonian $H_{0}=-\frac{1}{2}\nabla^{2}+V(r)$ can be separated as

$$\psi_{\mathrm{nlm}}(\vec{r})=R_{nl}(r)Y_{lm}(\theta ,\varphi ),$$ (4)

where *n*, *l*, and *m* are the principal, angular momentum, and magnetic quantum numbers, respectively, and $Y_{lm}(\theta ,\varphi )$ is a spherical harmonic function.

Substituting $\psi_{\mathrm{nlm}}(\vec{r})$ into Eq. (1), we can get the radial Schrödinger equation as follows:

$$[-\frac{1}{r^{2}}\frac{d}{dr}(r^{2}\frac{d}{dr})+\frac{l(l+1)}{r^{2}}+V(r)]R_{nl}(r)=E_{nl}R_{nl}(r).$$ (5)

Here, we can introduce an angular momentum dependent model potential with the orbital angular momentum *l* of the valence electron, i.e., using *V_l_*(*r*) to replace *V*(*r*). To precisely describe the bound states in helium, we choose the model potential *V_l_*(*r*) constructed in Ref. 48. The radial wave function $R_{nl}(r)$ can be expanded as a linear combination of B-spline functions,

$$R_{nl}(r)=\sum_{i}D_{i}B_{i,k}(r).$$ (6)

Once the numerical form of $R_{nl}(r)$ is obtained,^49,50^ the TDM between two bound states can be calculated as

$$\mu =\langle R_{nl}(r)Y_{lm}(\theta ,\varphi )|\vec{r}|R_{n\prime l\prime }(r)Y_{l\prime m\prime }(\theta ,\varphi )\rangle .$$ (7)

Here, the magnetic quantum number *m* = m′ = 0 due to the linearly polarized laser, and the bound state is labeled by the quantum numbers *n* (n′) and *l* (l′).

In atomic physics, the oscillation strength is a common physical quantity to reflect the characteristics of bound states and can be measured experimentally, which is proportional to the square of TDM,^44,51^ and thus, it is always a positive value. However, the TDM calculated in Eq. (7) has the positive or negative sign, to be called as the “phase” alternatively, which is often ignored. We will shed light on its role in the calculation of ATA spectrum in this paper.

### B. Single-atom response obtained with the multilevel model

**FIG. 1.**
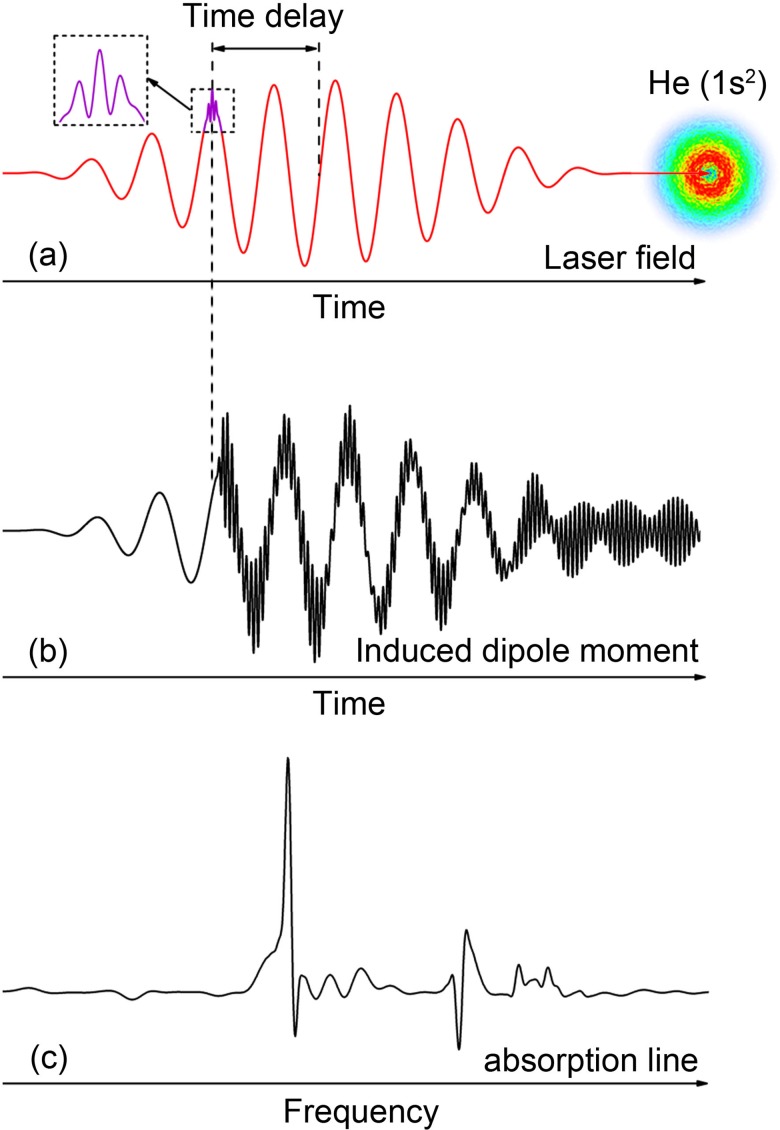
Schematic diagram of calculating the absorption line of helium at a fixed time delay. (a) Infrared laser (red) and time-delayed extreme ultraviolet attosecond pulse (purple) acting on the ground-state helium. (b) Time dependent induced dipole generated by the combined laser field in (a). (c) The absorption line obtained by transforming the dipole moment in (b) into the frequency domain.

To obtain the response of the atom, interacting with the XUV and IR laser pulses at a fixed time delay as shown in Fig. 1(a), we need to solve the TDSE of the electron as follows:

$$i\frac{\partial }{\partial t}\Psi (t)=(H_{0}+H_{1})\Psi (t),$$ (8)

where *H*_0_ is the field-free Hamiltonian and $H_{1}=-zE_{\mathrm{laser}}(t)$ describes the interaction of the atom with IR laser and delayed XUV pulse within the dipole approximation and the length gauge. Equation (8) can be accurately solved by using some numerical methods^52^ or can be approximately solved by truncating the basis set and by including a few bound states only. The latter is called the multilevel model. Within this model, the time-dependent wave function of the electron can be expanded by using the eigenstates of the undressed atomic system,

$$\Psi (t)=\sum_{l=0}^{l_{\text{max}}}\sum_{n=1}^{n_{\text{max}}}C_{n,l}(t)e^{-i\omega_{n,l}t}|n,l\rangle ,$$ (9)

with *n_max_* and *l_max_* being the largest principal and angular momentum quantum numbers of atomic states. The Dirac symbol |n,l⟩ represents the eigenstate, and $C_{n,l}(t)$ and $\omega_{n,l}$ are the time-dependent coefficient and eigenenergy of the electronic state, respectively.

Substituting Eq. (9) into Eq. (8), we can get a set of coupled equations,

$$i{\dot{C}}_{i}(t)=-E_{\mathrm{laser}}(t)\sum_{i\neq j}\mu_{ij}C_{j}(t)e^{i(\omega_{i}-\omega_{j})t},$$ (10)

where the subscripts *i* and *j* represent two states with different quantum numbers (*n*, *l*) and *μ_ij_* is the TDM, which can be calculated by Eq. (7). Then, the laser induced dipole moment *d*(*t*) as shown in Fig. 1(b) is obtained by

$$d(t)=\sum_{i\neq j}\mu_{ij}C_{i}^{*}(t)C_{j}(t)e^{i(\omega_{i}-\omega_{j})t}+c.c.,$$ (11)

where *c.c.* means the complex conjugate.

To describe the modulation of the delayed XUV field interacting with an IR field on the dressed atom, the single-atom response function is given directly (see Refs. 34 and 53 for details) as follows:

$$\tilde{S}(\omega )=-2Im[\tilde{d}(\omega )\tilde{\varepsilon^{*}}(\omega )],$$ (12)

where $\tilde{d}(\omega )$ and $\tilde{\varepsilon }(\omega )$ are the Fourier transforms of the time-dependent induced dipole and the combined electric field, respectively. Number 2 represents two electrons in the helium atom. To make the induced dipole moment *d*(*t*) smoothly going to zero when the laser pulses are off, it is multiplied by a window function in the form of $W(t,\tau )={\text{cos}}^{2}(\pi (t-\tau )/2T_{2})$, in which *T*_2_ is called the dephasing time. By using Eq. (12), we can calculate the absorption line at a fixed time delay of two laser pulses, as shown in Fig. 1(c).

## III. RESULTS AND DISCUSSION

In this section, we will first present the single-atom ATA spectra by using the multilevel model described in Sec. II B, calibrated with the TDSE results. Next, we will reveal why the phase of TDM is crucial in the multilevel model. Then, we will discuss the flexibility of choosing the atomic states in the multilevel model to analyze the specific structures in the absorption spectra. The dependence of the absorption spectrum on IR laser intensity is examined at last. Since we are under the SAE approximation, it is okay to only label one electron for convenience.

### A. The single-atom ATA spectra from different methods

In the calculations, the energy of the ground state is taken as zero, and the laser parameters used are as follows. The wavelength of the IR pulse is 800 nm, with the full-width-at-half-maximum (FWHM) of 10.67 fs and a peak intensity of 3 × 10^12^ W/cm^2^. Its envelope is cosine squared. The XUV pulse is assumed to have a Gaussian profile, with the FWHM of 400 as, centered at the photon energy of 22 eV, and its peak intensity is 1 × 10^11^ W/cm^2^. The initial electronic population is 1 for the ground state and 0 for the excited states. The dephasing time in the calculation is taken as 4 times of the FWHM of IR, and a longer *T*_2_ does not change the main structures in the ATA spectra.

**FIG. 2.**
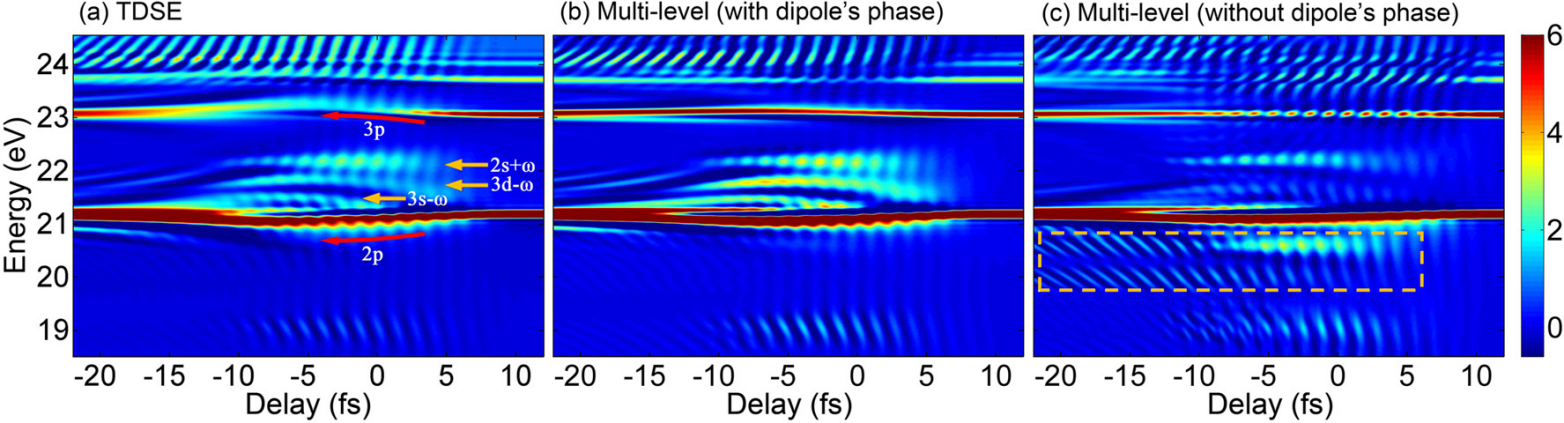
The ATA spectra of helium calculated by using three different methods: (a) the TDSE and the multilevel model (b) including or (c) ignoring the TDP. The same model potential is used in the three methods. In (a), the AC Stark shifts (red curved arrows) and the LISs (yellow straight arrows) are labeled. In (b) and (c), 20 energy levels are used with the principal quantum number *n* = 1∼6 and the angular momentum quantum number *l* = 0∼4. The irregular absorption structures are marked by a yellow dotted rectangle in (c).

The ATA spectra of the He atom as a function of time delay between the XUV and IR pulses calculated by the TDSE^52^ are shown in Fig. 2(a). The positive delay corresponds to the attosecond pulse arriving after the IR-dressing field with respect to its center. Some features can be easily observed in the ATA spectra as follows:^34^ (i) The AC Stark shift appears in the *p* state in the overlapping region of the XUV and IR pulses, for instance, in 2*p* and 3*p* states as labeled, (ii) several LISs are presented in the overlapping of two pulses (labeled in the figure), like 2*s* + *ω*, 3*d* - *ω*, and 3*s* - *ω* (∼22 eV) between the 2*p* and 3*p* absorption lines, and 2*s* - *ω* (∼19 eV) below the 2*p* absorption line, where *ω* is the angular frequency (or photon energy in a.u.) of the IR laser, standing for one photon, (iii) hyperbolic sideband structures can be seen at large negative delays near the *p*-state absorption lines, and (iv) the Autler-Townes splitting starts to occur at the small negative delay for *p*-state absorption lines. Note that these features are similar to those in Refs. 54 and 55 under similar laser parameters.

In the multilevel model to calculate ATA spectra for the He atom, 20 electronic states (*n *≤* *6 and *l *≤* *4) are used, which are already enough to give the converged spectra in the interested spectral region. We choose to maintain the phase (or the ± sign) of TDM calculated in Eq. (7) or neglect it. Note that the TDP has been commonly ignored before in the three-level model because the transition dipole was deduced from the oscillator strengths and its phase cannot be determined. We can see that by including the dipole phase, the ATA spectra in Fig. 2(b) are very similar to those in Fig. 2(a), and all important features discussed previously in the TDSE spectra can be quite nicely reproduced including the presence of the AC Stark shift, LISs, hyperbolic sidebands, and Autler-Townes splitting. In contrast, without the dipole phase, the ATA spectra in Fig. 2(c) are quite different from those either in Fig. 2(a) or in Fig. 2(b). In particular, the AC Stark shift of the 3*p* state becomes invisible, some LISs, such as 3*d* - *ω* and 3*s* - *ω*, are missing, and two irregular absorption structures are obviously presented right below the 2*p* absorption line. In the following, we will explain why the dipole phase plays an important role in the ATA and under what conditions it can be neglected.

### B. The role of the transition dipole phase in ATA processes

**FIG. 3.**
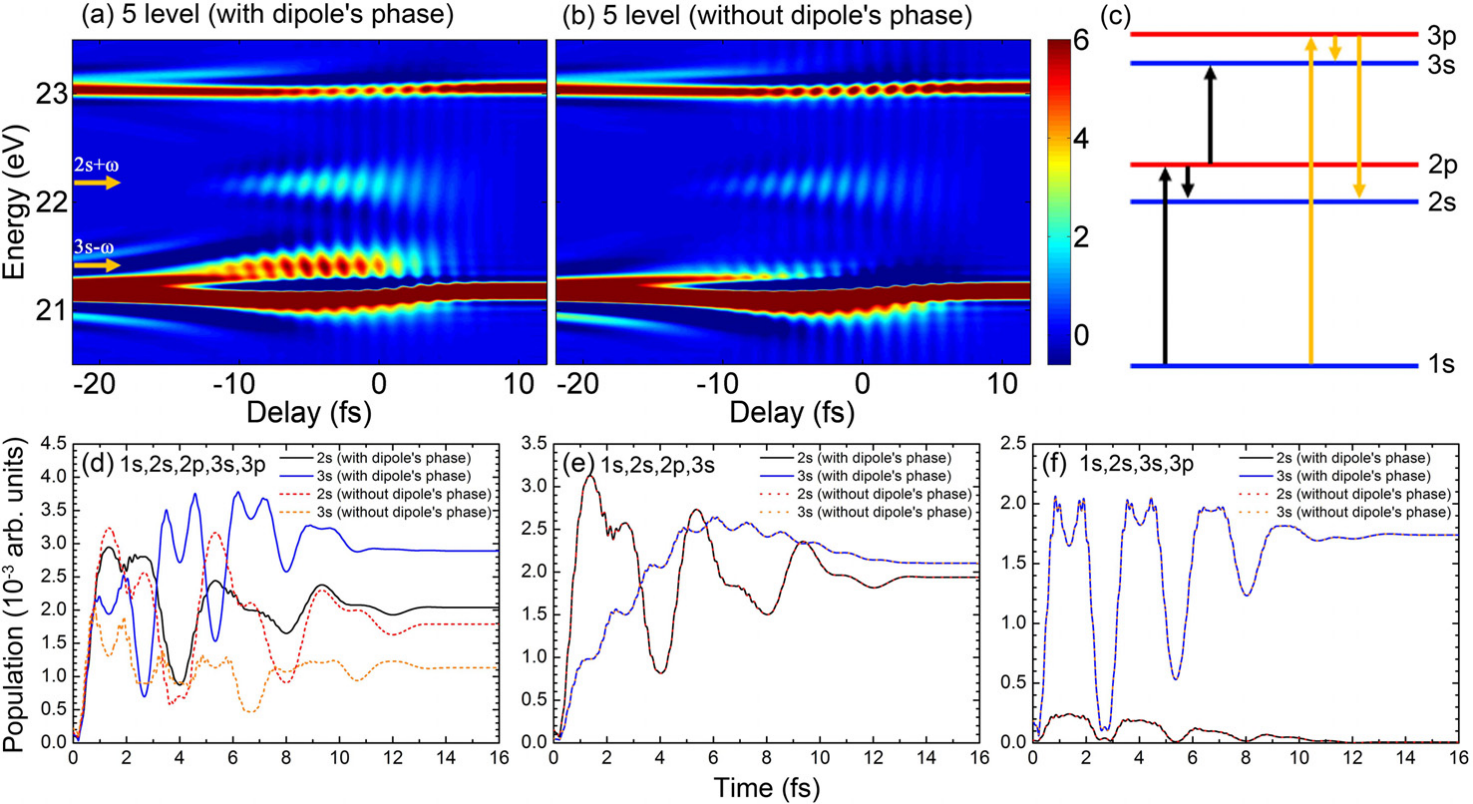
The ATA spectra of helium calculated by including 5 bound states in the multilevel model (a) with or (b) without the TDP. A sketch of the 5-level system is shown in (c). The two transition paths from the 1*s* to 2*s* or 3*s* state are indicated by black and yellow arrows. (d) The population of 2*s* and 3*s* states as a function of time when the time delay between the XUV and IR pulses is fixed as 0. A similar time-dependent population to those in (d) is plotted for (e) the 3*p* or (f) the 2*p* state removed from the 5-level system.

To simplify the illustration, we only include five bound states (1*s*, 2*s*, 2*p*, 3*s*, and 3*p*) in the multilevel model to calculate the ATA spectra of the He atom. The results are shown in Figs. 3(a) and 3(b) for including and ignoring the TDP, respectively. It is obvious that the behaviors of two LISs in the overlapping region between 2*p* and 3*p* absorption lines are quite different in two figures. Without the dipole phase, the 2*s* + *ω* state becomes weaker, and the 3*s* - *ω* state is almost gone. Since these LISs are closely related to the bound states of 2*s* and 3*s*, we thus check how these two states be excited and populated. As illustrated in Fig. 3(c), there are two paths for the electron excited from the ground state 1*s* to 2*s* (or 3*s*) according to the one-photon transition rule. One is through the 2*p* state: 1*s* → 2*p* → 2*s* (or 3*s*), and the other is through the 3*p* state: 1*s* → 3*p* → 2*s* (or 3*s*). In Fig. 3(d), we calculate the population of 2*s* and 3*s* at the time delay between the XUV and IR pulses as 0. The previously discussed behaviors of LISs in the ATA spectra can be traced back to the time-dependent population of the 2*s* (or 3*s*) state. It shows that the population of the 2*s* state follows a similar trend with the time with or without the dipole phase, while the significant differences can be seen in the time-dependent population of the 3*s* state.

To further explore the role of dipole phase, we remove one atomic state (2*p* or 3*p*) in the multilevel model; thus, only one path [yellow or black arrows in Fig. 3(c)] from 1*s* to 2*s* (or 3*s*) is left. We again check the population of 2*s* and 3*s* states in the 4-level system. As shown in Figs. 3(e) and 3(f), with or without the dipole phase, the population of the 2*s* (or 3*s*) state is unchanged when either the 2*p* or 3*p* state is removed. We have checked the ATA spectra with or without the dipole phase (not shown) in the 4-level system, and they are found to be identical. This indicates that the phase of the transition dipole is not important if only one path is allowed for the excited state and also shows the validity of the widely used three-level model without considering the TDP.

Therefore, we can conclude that if more than one path to excite the electronic state exists, the phase of the transition dipole is very essential to determine its correct time-dependent population and induced dipole moment, thus changing the absorption spectrum. With the increase in electronic states in the multilevel model, the interference between the multiple paths becomes very common and the TDP cannot be ignored.

### C. The selection of electronic states in the multilevel model

**FIG. 4.**
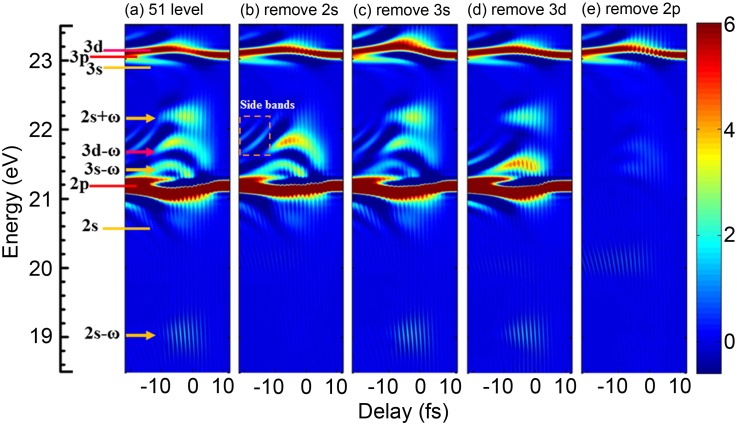
(a) The ATA spectra of helium calculated with the multilevel model by including 51 bound electronic states (*n *=* *1–11 and *l *=* *0–5). (b)–(d) The ATA spectra when the 2*s*, 3*s*, 3*d*, and 2*p* states are removed, respectively. The energy levels of a few eigenstates and light-induced states are labeled. The structure of sidebands is also marked (yellow dotted rectangle).

In the multilevel model, we can freely remove or add some bound states to analyze the interesting features in the ATA spectra. One example is to selectively take out an electronic state from the 51-level model (*n *=* *1–11 and *l *=* *0–5). Here, we use a large number of electronic states to ensure that there is no other state that could modify the ATA spectrum. The adsorption spectra calculated with 51 electronic states are shown in Fig. 4(a). When we remove 2*s*, 3*s*, and 3*d* states, respectively, the LISs of 2*s* ± *ω*, 3*s* - *ω*, and 3*d* - *ω* either disappear or become weakened in Figs. 4(b)–4(d), indicating that the LIS is closely related to individual electronic state. This conclusion is similar to that obtained with the Floquet theory.^34,55^ Note that the structure at 21.4 eV (overlapped with 3*s* - *ω*) in Fig. 4(a) is not completely suppressed by removing the 3*s* state in Fig. 4(c) because it is also contributed by the Autler-Townes splitting from the 2*p*-state absorption line. In Fig. 4(e), the 2*p* state is taken out, and obvious changes can be found compared to the ATA spectra in Fig. 4(a). All the LISs become invisible because the excitation of 2*s*, 3*s*, and 3*d* states from the 1*s* state is mainly through the 2*p* state. Moreover, the 2*p*-state absorption line with the AC Stark shift and its adjacent sidebands vanish.

Now, we can identify that specific electronic states are mostly responsible for the LIS or the *p*-state absorption lines in the ATA spectra. It can be understood qualitatively that the curved structures around a time delay of 0 displayed for all LISs and *p*-state absorption lines in Fig. 4(a) are due to the IR intensity dependent AC Stark shift. As the time delay changes from the negative to positive value, the IR intensity increases first and then decreases, and the AC Stark shift of each related electronic state follows the same tendency.

**FIG. 5.**
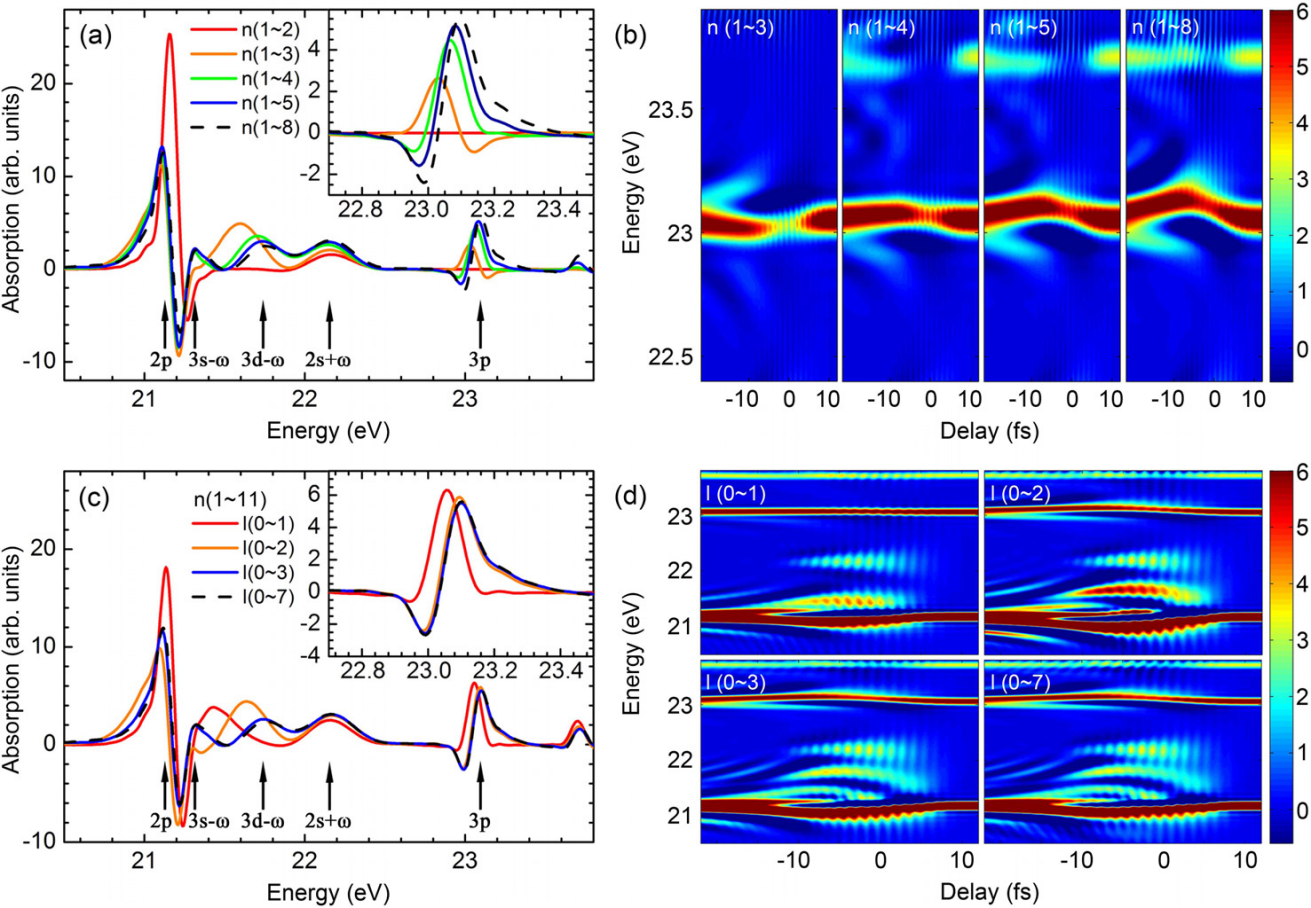
The evolution of the AC Stark shift [(a) and (b)] and laser induced states [(c) and (d)] with the increase in electronic states (labeled by quantum numbers *n* and *l*) in the multilevel model for the He atom system. In (a) and (c), the absorption lines are shown for the time delay between the XUV and IR pulses as 0, and the ATA spectra as a function of time delay are plotted in (b) and (d). In (a) and (b), the principle quantum numbers *n* are increased with all allowed angular momentum quantum numbers. In (c) and (d), the angular momentum quantum numbers *l* are increased, while the principle quantum numbers are fixed as *n *=* *1–11.

As shown in Fig. 4, the distinguishing features in ATA spectra between 18.5 and 23.5 eV mostly originate from 1*s*, 2*s*, 2*p*, 3*s*, 3*p*, and 3*d* states. How are they affected by the other electronic states? We employ two approaches to systematically add the bound states in the multilevel model to observe the changing trend of absorption lines and structures. One is to increase the principle quantum numbers *n* with all allowed angular moment quantum numbers *l* and the other is to increase the angular moment quantum numbers *l* while the quantum numbers *n* are fixed. The absorption lines at zero time delay are shown in Figs. 5(a) and 5(c). When *n *=* *1–3 or *l *=* *0–2 as labeled in the figures, all dominant states are included. First, we observe and analyze the changes in LISs. In Fig. 5(a), the absorption structures around the LIS of 3*d* - *ω* change significantly when *n* is increased from 3 to 4 (from yellow to green lines); a further increase in *n* only slightly changes the spectral shape. It is not clear which state plays a major role in such a rapid change, but the obvious candidates are 4*s*, 4*p*, 4*d*, and 4*f*. We then check Fig. 5(c). The first three states are included when *l *=* *0–2 (yellow line); however, the spectra of LISs are not converged until the *f* state is added (blue line). The ATA spectra with the time delay in Fig. 5(d) also show that the convergence is reached with the addition of *f* states. Thus, we can conclude that *f* states, especially the 4*f* state, are very important for the formation of LISs in the interested spectral region because of the strong coupling between *f* and *d* states, leading to a significant change in the population of *d* states.

Another attractive phenomenon in Figs. 5(a) and 5(c) is the change of the *p* state absorption line, especially the 3*p* state (see enlarged views in both figures). In Fig. 5(c), the absorption line is quickly converged if all *s*, *p*, and *d* states are included. However, in Fig. 5(a), the convergence with *n* is very slow. This can also be seen in the ATA spectra with the change of time delay in Fig. 5(b), especially for the AC Stark shift of the 3*p* state. It can thus be concluded that all *s* and *d* states even with higher *n* have the effects on the emerging of 3*p*-absorption line and its AC Stark shift due to the allowed transitions between *s* and *p* states and between *p* and *d* states.

### D. IR intensity dependent absorption spectrum: The multilevel model vs the TDSE calculations

**FIG. 6.**
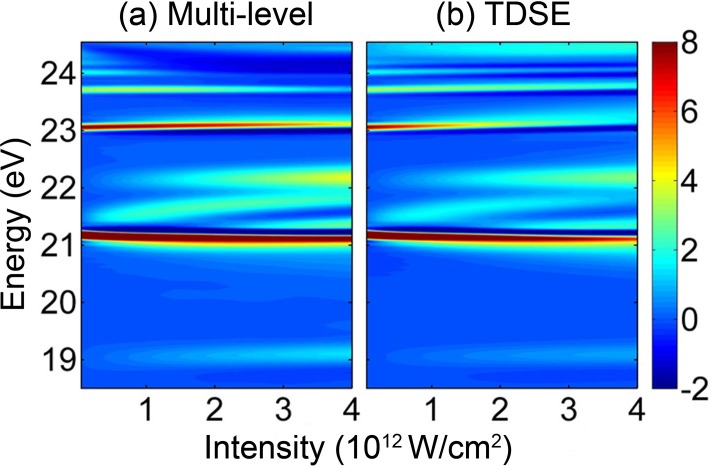
The ATA spectra of the He atom as a function of IR laser intensity when the time delay between XUV and IR pulses is fixed as 0, simulated by (a) the multilevel model and (b) the TDSE calculation.

In the above simulations, the peak intensity of the IR laser is fixed. It is interesting to see how the absorption spectrum changes with the IR intensity. This scheme has been demonstrated experimentally, which can be easily operated.^42^ We fix the time delay between the XUV and IR pulses at zero and calculate the absorption spectrum as a function of the IR laser intensity by using the multilevel model (51 electronic states included). As shown in Fig. 6(a), with the increase in IR intensity up to 4 × 10^12^ W/cm^2^, the 2*p*- and 3*p*-state absorption lines are shifted down and up, respectively, and all LISs between them move up. This can be expected from the dependence of the AC Stark shift of the electronic state on the IR intensity. For comparison, the TDSE method is also applied to calculate the ATA spectrum in Fig. 6(b). The simulated results by two methods agree very well when the energy is below 24 eV. Above 24 eV, the TDSE gives more pronounced and broadened structures with the increase in IR intensity. This is possibly due to the transitions between the bound and continuum states (or the electron ionization) in the TDSE, which is not considered in the current multilevel model.

## IV. CONCLUSIONS

In summary, we theoretically obtained the well-defined phase of the transition dipole moment between the bound atomic states by numerically solving the stationary Schrödinger equation with the B-spline basis set and proposed a modified multilevel model accounting for the phase of the transition dipole. We found that this phase often neglected previously plays an important role in accurate simulations of atomic ATA spectra driven by an attosecond XUV pulse and a delayed moderate IR field. Calibrated with the TDSE calculation, with the TDP, the multilevel model could reproduce almost all pronounced features in the ATA of atomic helium system, while this model generates irregular structures if the TDP is omitted. If more than one path contributes to the population of the excited state from the ground state, this phase significantly modifies the ATA spectra. By removing or adding specific electronic state according to the principle quantum numbers or the orbital angular moment quantum numbers, the multilevel model with the correct TDP is capable of analyzing the attractive structures in the ATA spectra, such as LISs, AC Stark shift of the absorption line, sideband structures, and so on. Furthermore, we checked the validity of our multilevel model by comparing with the IR-intensity dependent ATA spectra from the TDSE calculations.

It is worth mentioning that the multilevel model established in this work has certain limitations. For example, IR intensity should be kept low; otherwise, the significant ionization would be created, and the photon energy of the attosecond XUV pulse should be small enough to only excite one electron in helium without ionization or avoid the autoionization excited by two electrons.^10,13,14,24,36,43,56–58^ To simulate the experimentally measured ATA spectra and to reveal the ultrafast dynamics of bound electrons for atomic systems, the multilevel model needs to be continuously developed by further considering the macroscopic propagation,^16,37,53,59–61^ which will be carried out in our future work.
